# The 30 m vegetation maps from 1990 to 2020 in the Tibetan Plateau

**DOI:** 10.1038/s41597-024-03649-7

**Published:** 2024-07-20

**Authors:** Fan Wu, Hongrui Ren, Guangsheng Zhou

**Affiliations:** 1 Department of Geomatics, Taiyuan University of Technology, Taiyuan, 030024 China; 2 State Key Laboratory of Severe Weather, Chinese Academy of Meteorological Sciences, Beijing, 100081 China

**Keywords:** Environmental health, Ecosystem ecology

## Abstract

The Tibetan Plateau (TP) is crucial for global climate change and China’s ecological security. Given recent drastic changes in vegetation from climate change and human activities, long-term vegetation monitoring is urgently required. This study produced the vegetation maps of the TP from 1990 to 2020 every ten years using random forest classifier and Landsat imagery. We selected the same stable samples and features for mapping to reduce errors between years and proposed spatial filtering to further improve the accuracy. The overall accuracy surpassed 95.00%, with all Kappa coefficients exceeding 0.95. A further assessment based on sampling sites from literature and field survey was higher than 80%. The importance ranking results indicated that in the TP, climate factors and terrain factors are the most important factors in the vegetation mapping. This study provides a method for mapping vegetation in alpine areas and data support for researching the dynamic change of vegetation on the TP and evaluating its response to climate change.

## Background & Summary

Vegetation, which affects carbon cycling, water balance and energy exchange, is the crucial component of terrestrial ecosystem and the most sensitive part to climate change^1^. In recent decades, the spatial distribution of vegetation has undergone substantial modifications at both local and global scales, primarily attributable to the impacts of climate change and anthropogenic activities^2^. Therefore, the availability of timely, high-resolution vegetation maps is immensely important for climate and environment studies and sustainable development.

The advent of remote sensing technology and machine learning algorithms makes it possible to generate large scale and high-resolution land cover products^3,4^. Remote sensing data possess the capability of extensive spatial coverage and long-term series imagery, overcoming the inherent limitations of labour-intensive and time-consuming manual surveys^5^. Machine learning algorithms, notably random forest (RF) and support vector machine (SVM), integrated with remote sensing data, has become increasingly prevalent, and has achieved high classification accuracy^6^. Sulla-Menashe *et al*.^7^ used MODIS data and RF to produce global annual land cover products for 2001–2018. Yang *et al*.^8^ adopted Landsat data, terrain (slope, aspect and elevation), latitude and longitude to produce the annual land cover dataset of China during 1990–2019 using the RF classifier. However, deficiency exists in studies integrating climate, terrain, and remote sensing data for land use/cover and vegetation mapping.

The Tibetan Plateau (TP) is the largest and highest geographical unit in the world. The unique geographical location of the area, has given rise to varied climate zones and fostered diverse vegetation types, encompassing alpine meadow, alpine grassland, alpine desert, and alpine vegetation^9^. In the last five decades, the TP has undergone a warming rate twice as rapid as the global average. Simultaneously, there is a trend of increased precipitation^10^. Consequent to climatic change, the ecosystem of the TP has undergone profound changes, such as the degradation of permanent snow/ice^11^. Nevertheless, owing to the intricate geographical environment, an apparent spatial heterogeneity exists within the impact mechanism between vegetation and climate. This intricacy directly engenders controversy regarding the trend and process of vegetation change under the influence of climate change^12^. Therefore, a comprehensive investigation into vegetation changes is vital for understanding the spatial arrangement of vegetation and gaining insights into the impacts of climate change on vegetation dynamics.

The earliest record of vegetation for the TP is the vegetation map of China (1:1,000,000)^13^. This map depicts the vegetation distribution during the 1980s–1990s. However, currently, noticeable changes have occurred in the vegetation on the TP^14^. In recent years, there have also been studies used remote sensing data to obtain vegetation distribution maps on the TP. Zhang *et al*.^15^ collected seven vegetation distribution/land cover products produced with different data sources and different classification methods. Taking full advantage of these products and employing an integrated approach, a vegetation map for the TP in 2020 was produced. Zhou *et al*.^16^ made a vegetation map of the TP in 2020 using Sentinel-2A/B remote sensing images, terrain and climate data. Furthermore, there are global land use products like GlobeLand30^17^. However, these classification systems primarily focus on land use types and do not provide sufficient detail to accurately capture the unique vegetation characteristics of the TP. Therefore, there is still a need for high-resolution, long-term vegetation distribution maps for the TP.

To address this problem, this study developed a vegetation classification system in the TP. By integrating remote sensing with climate and terrain data, we produced vegetation maps of the TP during 1990–2020. Based on the field survey experience, stable samples were selected. Then, long-time series Landsat remote sensing images were combined with climate and terrain data to construct 153 features. By employing RF classifier, we obtained an optimal feature set for all years. The vegetation mapping was completed by using the optimal feature set and samples with the RF classifier, followed by applying spatial filtering post-processing method. We used this dataset to study the vegetation change trend and process over the past 30 years, and analysed variations in patterns among different vegetation types. The results provide fundamental data to comprehend the correlation between vegetation dynamics and climate change.

## Methods

### Study area

The Tibetan Plateau (25° 59′ 30″ N~40° 1′ 0″ N, 67° 40′ 37″ E~104° 40′ 57″ E) is located in central Asia, spanning nine countries, including China, India, Pakistan, and others^18^. Influenced by westerly circulation and the Indian monsoon, the annual precipitation on the TP exhibits a gradient, decreasing from approximately 2000 mm in the southeast to around 20 mm in the northwest. Concurrently, temperature displays a corresponding decrease with increasing altitude and latitude, ranging from 20 °C in the southeast to −10 °C in the northwest. The spatial variability in temperature and precipitation significantly influences the vegetation pattern on the TP, contributing to its intricate and diverse array of vegetation types. Along the southeast to northwest gradient, the TP exhibits vegetation zones encompassing forest, scrub, alpine meadow, alpine grassland, and alpine desert, among others. And alpine ecosystems exhibit a pronounced sensitivity and vulnerability to external influences^19^. The study area is the TP in China, which includes Tibet, Qinghai, Gansu, Sichuan, Yunnan and Xinjiang, with an expansive area of approximately 2.6 million square kilometers. This region is distinguished by an average elevation exceeding 4000 meters.

### Data

#### Visually interpret stable sample data

Considering the vegetation distribution in the TP^13^, as well as the field survey, the classification system in this study includes 16 vegetation types: evergreen broad-leaved forest, evergreen coniferous forest, coniferous and broad-leaved mixed forest, deciduous broad-leaved forest, deciduous coniferous forest, scrub, alpine scrub meadow, alpine meadow, alpine grassland, alpine vegetation, alpine desert, cultivated vegetation, wetland, water, permanent snow/ice and non-vegetated area.

**Fig. 1:**
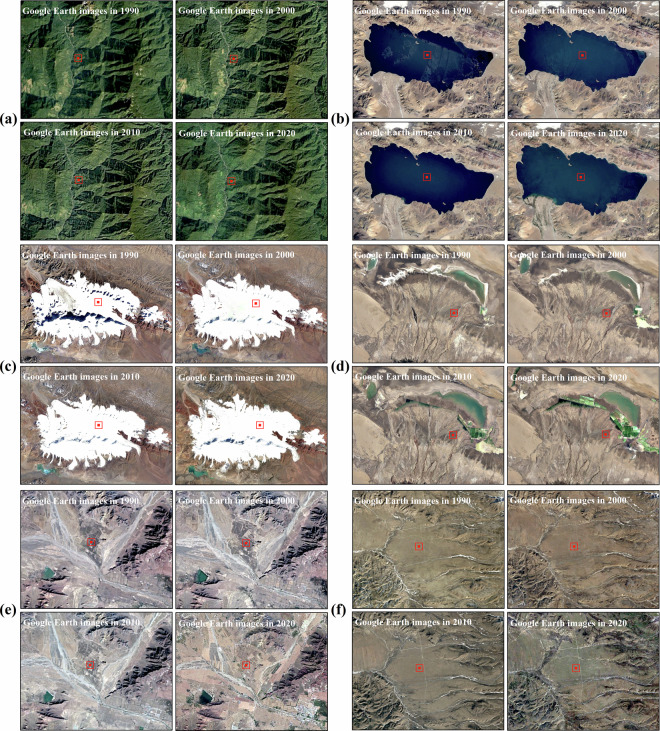
Examples of stable samples in the TP over the past 30 years.

**Fig. 2:**
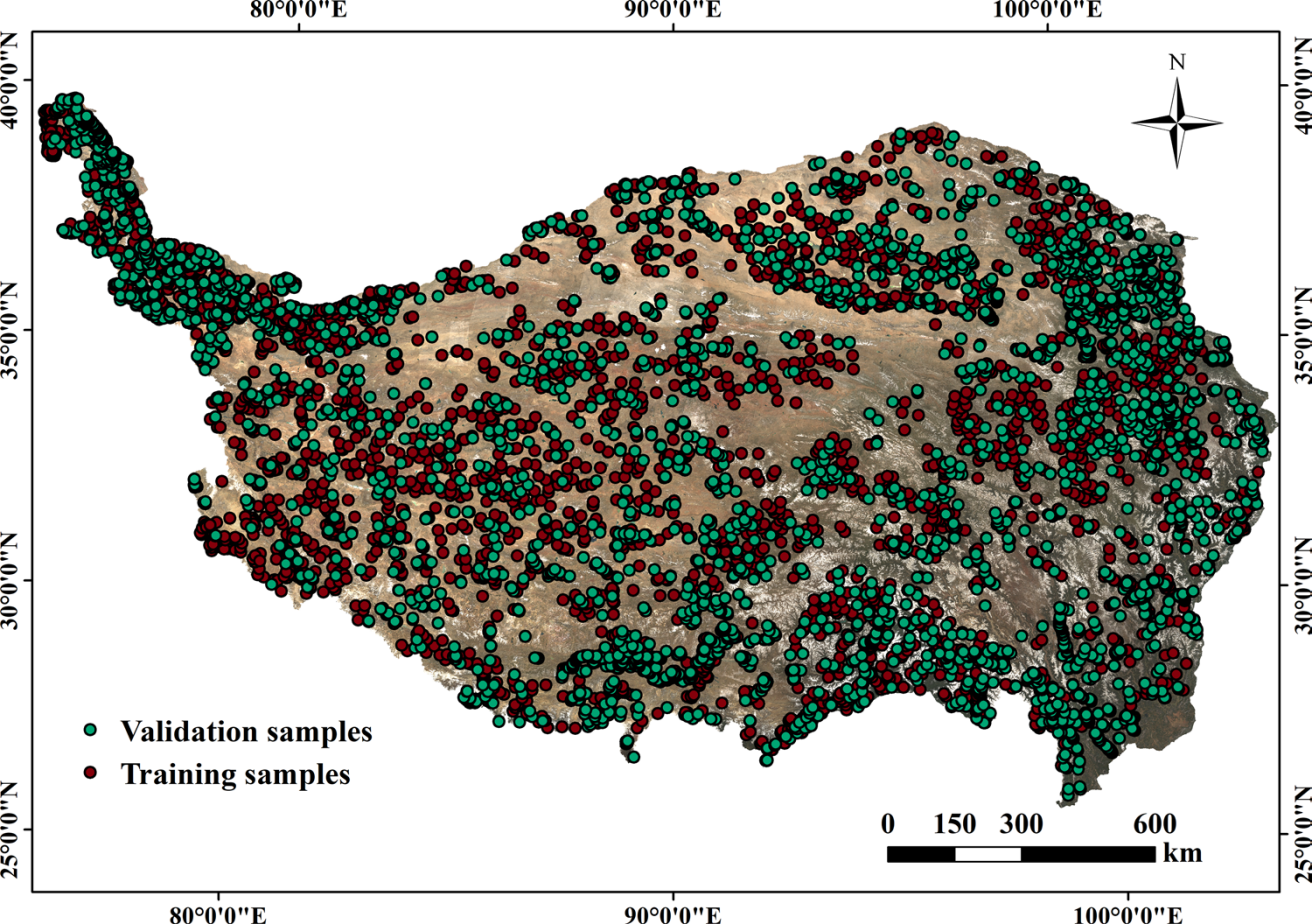
Distribution of training and validation samples in vegetation mapping on the TP.

Our team conducted field trips on the TP in August 2019, August 2020 and September 2021. Based on field survey experience and visual interpretation of Google Earth, 11733 stable samples were selected in this study. Specifically, remote sensing images from 1990 to 2020 were considered when select the samples, ensuring that the type of vegetation at each selected samples remained the same throughout all periods to enhance the reliability of samples. Figure 1 represents sampling sites where the vegetation types have remained unchanged over a span of 30 years, including evergreen broad-leaved forest (a), water (b), permanent snow/ice (c), wetland (d), cultivated vegetation (e) and alpine meadow (f). According to the ratio of 3:7, the selected samples were segregated using stratified random sampling method, resulting in 8213 training samples and 3520 validation samples, as presented in Table 1. The samples were chosen to be distributed as evenly as possible, with an intentional augmentation of sample density in regions characterized by intricate vegetation types (Fig. 2).

**Table 1:** Number of training and validation samples in vegetation mapping on the TP.

Vegetation type	ID	Training samples	Validation samples	Total
Evergreen broad-leaved forest	1	368	148	516
Evergreen coniferous forest	2	411	187	598
Coniferous and broad-leaved mixed forest	3	127	42	169
Deciduous broad-leaved forest	4	508	230	738
Deciduous coniferous forest	5	208	94	302
Scrub	6	557	236	793
Alpine scrub meadow	7	212	92	304
Alpine meadow	8	1090	472	1562
Alpine grassland	9	814	323	1137
Alpine vegetation	10	352	103	455
Alpine desert	11	599	244	843
Cultivated vegetation	12	790	329	1119
Wetland	13	395	178	573
Water	14	1004	493	1497
Permanent snow/ice	15	650	301	951
Non-vegetated area	16	128	48	176
Total		8213	3520	11733

#### Satellite images

Remote sensing data from Landsat, a satellite series initiated by the National Aeronautics and Space Administration (NASA), have been obtained. Given the considerable uncertainties arising from the suboptimal quality of remote sensing imagery in 1980. This study initiated vegetation distribution mapping from the year 1990, producing maps at ten-year intervals. Considering the limited availability of data in the earlier years, some regions had incomplete data coverage, Landsat 5 TM images from 1990 to 1991 were selected for vegetation mapping in 1990. Using Landsat 7 ETM + to produce the vegetation maps in 2000 and 2010. Images from 2000 to 2001 were used for 2000, and in 2010 only images from 2010 were used. Landsat 8 OLI surface reflectance data spanning the entire duration of 2020 were employed to generate a vegetation map. In this study, six bands-blue, green, red, near red, shortwave infrared 1, and shortwave infrared 2-were used for vegetation mapping, with a resolution of 30 m.

#### Climate and terrain data

Climate and terrain data were combined with remote sensing data in this study. Climate data include annual minimum temperature, annual maximum temperature, total precipitation, and average temperature for the year and four seasons. These data, representing extreme values, annual trends, and seasonal environmental factors, were calculated based on monthly precipitation and average temperature published by Peng *et al*.^20–24^. The data had a resolution of 1000 m and was automatically resampled to 30 m using bicubic method. The Shuttle Radar Topography Mission (SRTM) data and variables derived from it were used as terrain factors in this study to better capture topographic changes, with a resolution of 30 meters.

#### Validation data

**Fig. 3:**
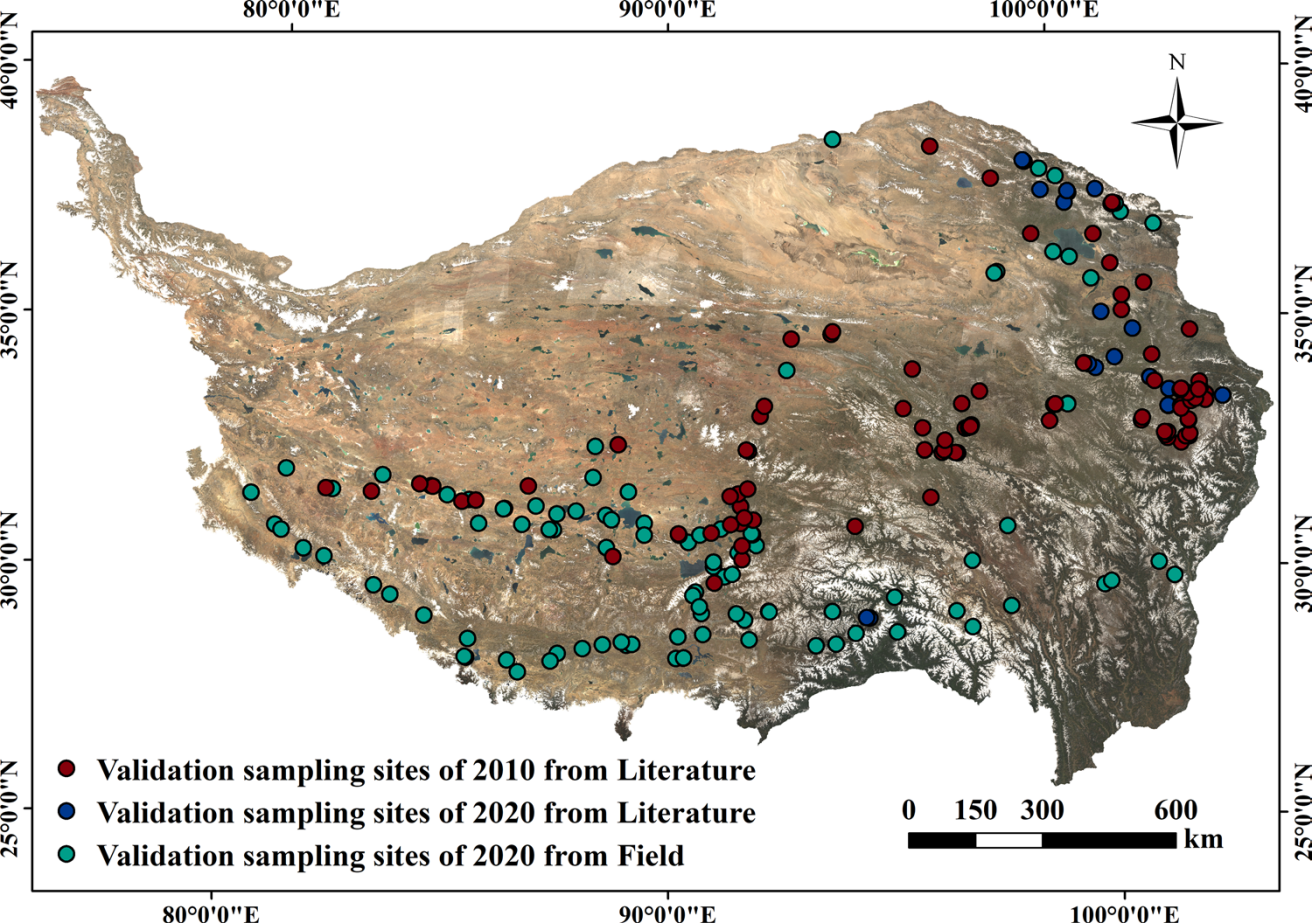
Distribution of validation sampling sites obtained from literature and field survey for vegetation mapping on the TP.

Apart from the visually interpreted validation samples, we collected 176 sampling sites from the TP field survey to thoroughly assess the quality of our results. To further validate the classification accuracy in this study, sampling sites representing vegetation type on the TP were extracted from the published literature in the Web of Science database and the China National Knowledge Infrastructure (CNKI). Firstly, search for keywords such as “vegetation of Qinghai-Tibet Plateau” and “vegetation of TP”. The retrieved results are then filtered. The screening criteria were as follows: geographical location within the TP and includes accurate sampling time, location, and vegetation type. Finally, A total of 38 eligible literature (Supplementary File 1) and 173 sampling sites were found. This study verifies the accuracy of the maps for the years 2010 and 2020. The data from 1990 and 2000 are relatively antiquated, with a limited number of sampling sites and challenges in ensuring data quality. Using the sampling sites from the years 2009 to 2011 as the validation data for the year 2010, there are a total of 114 sampling sites, including 98 sampling sites in alpine meadow, 13 sampling sites in alpine grassland, 1 sampling site in alpine vegetation, and 2 sampling sites in alpine desert. Leveraging sampling sites collected between the years 2019 to 2021 as the validation dataset for the year 2020, there are a total of 59, all of which belong to the alpine meadow. In summary, there are 114 validation sampling sites for the year 2010 and 235 for the year 2020 (Fig. 3).

### Vegetation mapping of the TP

**Fig. 4:**
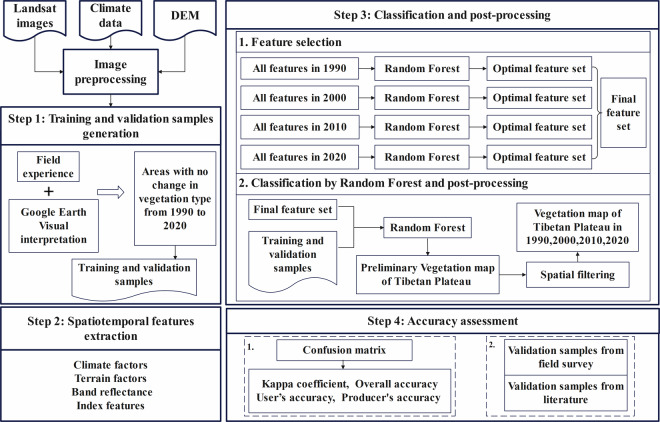
Vegetation mapping method for the TP.

The processing chain comprised four components: the generation of training and validation samples, construction of spatiotemporal features, classification and post-processing, and accuracy assessment (Fig. 4). Firstly, stable samples were selected in Google Earth by insights gained from field surveys. The features were then constructed using Landsat imagery combined with climate and terrain data. Subsequently, the optimal subset of features in each year is obtained. Specifically, the stable samples and all features were applied in a RF classifier, and the optimal subset of features was selected based on the calculated feature importance metrics and out-of-bag errors. By unifying the four feature sets, we got one feature set with good performance. Afterwards, the RF classifier was used to combine the stable samples and specified feature set for vegetation mapping. Spatial filtering post-processing was applied to refine these vegetation maps and generate the ultimate vegetation distribution map. Finally, the accuracy was assessed by the visually interpreted independent validation samples and sampling sites obtained from field surveys and published literature.

#### Spatiotemporal features construction

Features constructed in this study include four classes: climate factors, terrain factors, band reflectance and index features. Among them, climate factors include annual minimum temperature, annual maximum temperature and, summed precipitation and average temperature for the year and four seasons, which representing extreme, annual trends or seasonality environmental factors (Table 2). Terrain factors comprise elevation, slope and aspect. Band reflectance features contain red, green, blue, near infrared, short wave infrared 1 and short wave infrared 2 bands. Additionally, based on the above bands, 17 index features were constructed (Table 3). For both band reflectance features and index features, this study, leveraging time series data, sorted 23 features of each pixel within the selected time range. Subsequently, features were composited into the 15th, 30th, 45th, 60th, 75th and 90th percentiles. Time series percentiles decompose an irregularly distributed time series into a decreasing number of fixed features, which is more convenient for classification^25^. In total, there were 153 features for vegetation mapping of the TP from 1990 to 2020.

**Table 2:** Climate features of vegetation mapping.

Name	Abbreviation	Unit
annual precipitation	Pre	mm
annual average temperature	Tmp	°C
annual minimum temperature	Tmn	°C
annual maximum temperature	Tmx	°C
spring precipitation	Pre1	mm
summer precipitation	Pre2	mm
autumn precipitation	Pre3	mm
winter precipitation	Pre4	mm
spring average temperature	Tmp1	°C
summer average temperature	Tmp2	°C
autumn average temperature	Tmp3	°C
winter average temperature	Tmp4	°C

**Table 3:** Index features of vegetation mapping.

Name	Abbreviation	Formula	Sources
Normalized Difference Vegetation Index	NDVI	$\frac{NIR-\mathrm{Re}{\rm{d}}}{NIR+\mathrm{Re}d}$	^49^
Difference Vegetation Index	DVI	$NIR-\mathrm{Re}d$	^50^
Ratio Vegetation Index	RVI	$NIR/\mathrm{Re}d$	^51^
Soil Adjusted Vegetation Index	SAVI	$\frac{1.5\times (NIR-\mathrm{Re}d)}{NIR+\mathrm{Re}d+0.5}$	^52^
Enhanced Vegetation Index	EVI	$\frac{2.5\times (NIR-\mathrm{Re}{\rm{d}})}{NIR+6\times \mathrm{Re}d-7.5\times Blue+1}$	^53^
Renormalized Difference Vegetation Index	RDVI	$\frac{NIR-\mathrm{Re}d}{\sqrt{NIR+\mathrm{Re}d}}$	^54^
Vegetation Near-Infrared Reflectance Index	NIRv	$\frac{NIR\times (NIR-\mathrm{Re}d)}{NIR+\mathrm{Re}d}$	^55^
Green Chlorophyll Vegetation Index	GCVI	$\frac{NIR}{Green}-1$	^56^
Optimized Soil-Adjusted Vegetation Index	OSAVI	$\frac{1.16\times (NIR-\mathrm{Re}d)}{NIR+\mathrm{Re}d+0.16}$	^57^
Modified Simple Ratio	MSR	$\frac{NIR/\mathrm{Re}d-1}{\sqrt{NIR/\mathrm{Re}d+1}}$	^58^
Non-Linear Index	NLI	$\frac{NI{R}^{2}-\mathrm{Re}d}{NI{R}^{2}+\mathrm{Re}d}$	^59^
Normalized Difference Water Index	NDWI	$\frac{Green-NIR}{Green+NIR}$	^60^
Modified Normalized Difference Water Index	MNDWI	$\frac{Green-Swir1}{Green+Swir1}$	^61^
Normalized Difference Glacier Index	NDGI	$\frac{Green-\mathrm{Re}d}{Green+\mathrm{Re}d}$	^62^
Bare Soil Index	BI	$\frac{(Swir1+\mathrm{Re}d)-(NIR+Blue)}{(Swir1+\mathrm{Re}d)+(NIR+Blue)}$	^63^
Normalized Difference Built-up Index	NDBI	$\frac{Swir1-NIR}{S{\rm{w}}ir1+NIR}$	^64^
Index-based Built-up Index	IBI	$\frac{NDBI-(SAVI+MNDWI)/2}{NDBI+(SAVI+MNDWI)/2}$	^65^

#### Random forest classifier

The random forest (RF) classifier is a machine learning algorithm which is composed of multiple classification and regression decision trees. It selects random attributes while training decision trees, so as to increase the heterogeneity among classification models and enhance the generalization and prediction ability of the model^26^. It is widely employed in large-scale land cover/use mapping owing to its numerous advantages, including the capacity to manage high-dimensional data, the resilience to sample errors, and the stability in the face of missing data^8^. This study employs the RF classifier in Google Earth Engine (GEE) for modeling.

#### Feature importance evaluation and optimization

Feature importance was calculated by the RF classifier using the principle of average impure reduction. The features were sorted in descending order of importance, and then sequentially selected starting from the most important feature to the top two, top three, and so forth to obtain 153 feature sets. By computing the out-of-bag error, the feature set with the smallest out-of-bag error was chosen as the optimal feature set for the mapping.

#### Accuracy assessment

To quantitatively assess the accuracy of the vegetation maps produced in this study, two methods were employed. Firstly, a total of 3520 visually interpreted validation samples were used to calculate the confusion matrix, overall accuracy (OA), user’s accuracy (UA), producer’s accuracy (PA), and Kappa coefficient as evaluation criteria. The second method is to use the validation sampling sites obtained from the literature and field survey conducted by the research team.

1 $$UA=\frac{{X}_{ii}}{{\sum }_{i=1}^{n}{X}_{+i}}\times 100 \% $$

2 $$PA=\frac{{X}_{ii}}{{\sum }_{i=1}^{n}{X}_{i+}}\times 100 \% $$

3 $$OA=\frac{{\sum }_{i=1}^{n}{X}_{ii}}{N}\times 100 \% $$

4 $$Kappa=\frac{N{\sum }_{i=1}^{n}{X}_{ii}-{\sum }_{i=1}^{n}({X}_{i+}\times {X}_{+i})}{{N}^{2}-{\sum }_{i=1}^{n}({X}_{i+}\times {X}_{+i})}$$

Where *X*_*ii*_ denotes the count of validation samples accurately classified within a specific category; n is the overall count of categories; *X*_+__*i*_ represents the number of validation samples classified into the category; *X*_*i*+_ indicates the number of validation samples for this category; N is the number of validation samples.

#### Spatial filtering post-processing

To further enhance the reliability and accuracy of the classification results, post-processing operation was carried out on the preliminary vegetation distribution map of the TP. This method can correct discrete distributed pixels caused by misclassification^8^. Spatial filtering was performed within a 3 × 3 window. If the dominant classification within the window does not correspond to the central pixel type, it was identified as a classification error and rectified to align with the vegetation type exhibiting the highest proportion.

## Data Records

**Fig. 5:**
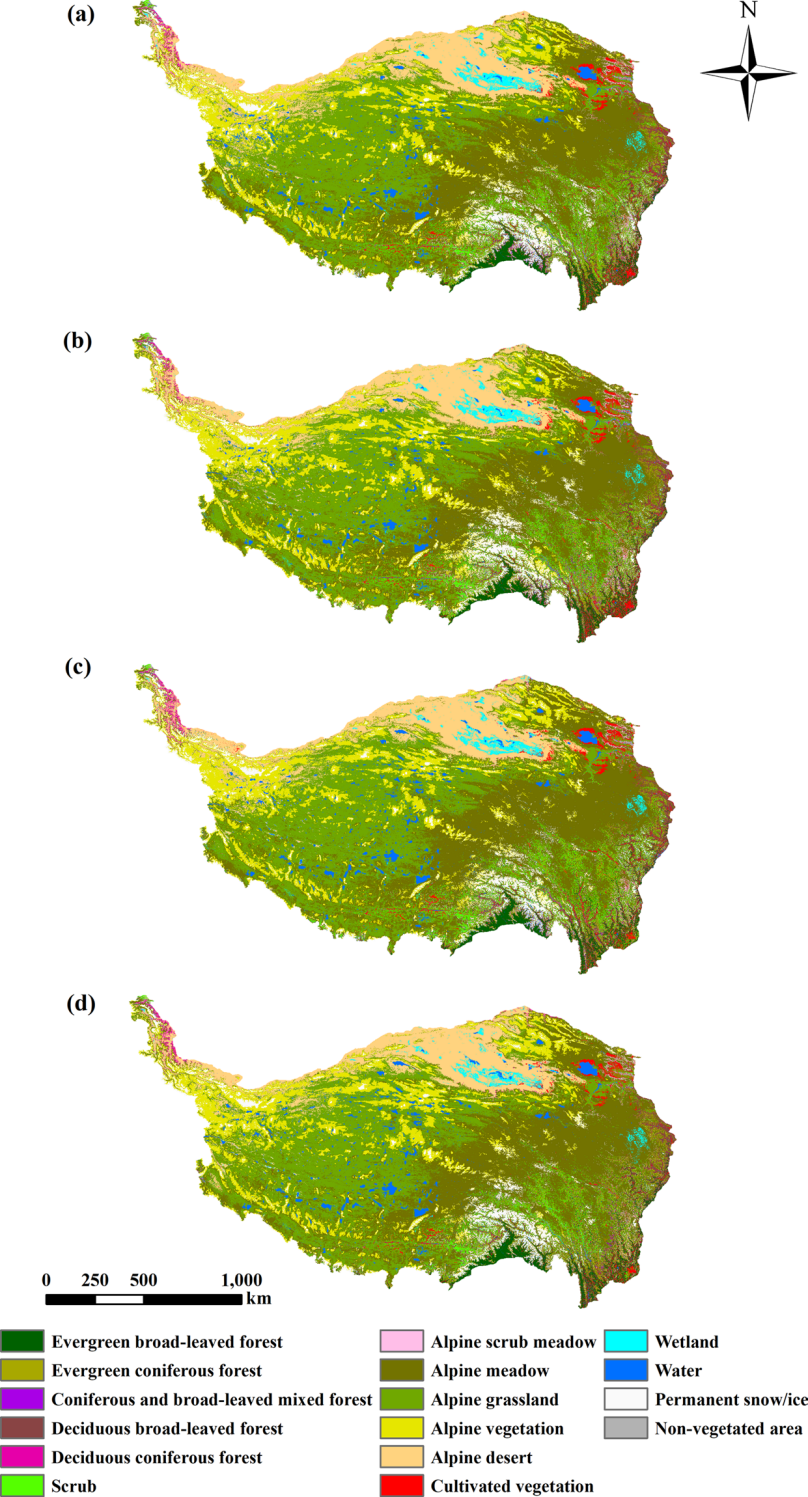
Vegetation distribution maps of the TP in 1990, 2000, 2010 and 2020.

The dataset generated in this study is available at the National Tibetan Plateau/Third Pole Environment Data Center 10.11888/Terre.tpdc.301126^27^. The dataset includes four years and sixteen categories, as shown in Fig. 5. The numbers 1 to 16 represent evergreen broad-leaved forest, evergreen coniferous forest, coniferous and broad-leaved mixed forest, deciduous broad-leaved forest, deciduous coniferous forest, scrub, alpine scrub meadow, alpine meadow, alpine grassland, alpine vegetation, alpine desert, cultivated vegetation, wetland, water, permanent snow/ice and non-vegetated area. The dataset is stored in TIFF format with a resolution of 30 meters. The file name is TPveg_yyyy.tif, where yyyy indicates the year. This data can be viewed through geographic information system software, such as ArcGIS.

## Technical Validation

### Classification accuracy of vegetation mapping on the TP

**Fig. 6:**
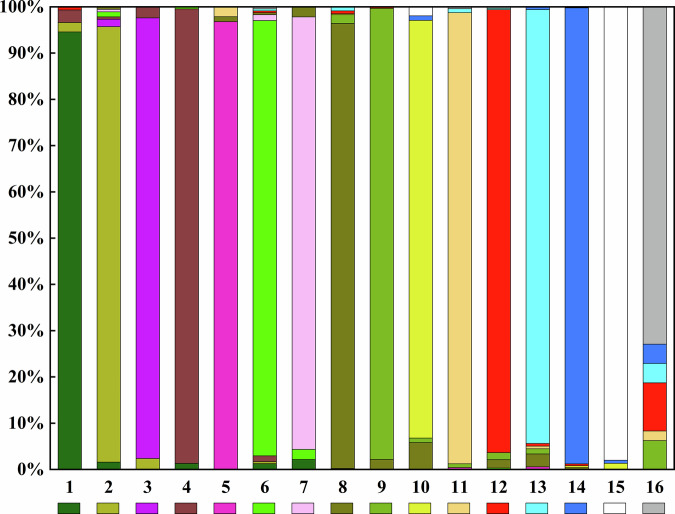
The confusion proportions for each vegetation types of the TP in 2020. The numbers 1 to 16 represent evergreen broad-leaved forest, evergreen coniferous forest, coniferous and broad-leaved mixed forest, deciduous broad-leaved forest, deciduous coniferous forest, scrub, alpine scrub meadow, alpine meadow, alpine grassland, alpine vegetation, alpine desert, cultivated vegetation, wetland, water, permanent snow/ice and non-vegetated area.

Based on all visually interpreted stable samples and 27 features, we generated the vegetation maps of the TP from 1990 to 2020. Derived from the 3520 visually interpreted validation samples, the OA of vegetation mapping on the TP from 1990 to 2020 were 95.74%, 95.88%, 95.54%, 95.99%, respectively. The Kappa coefficients were 0.95, 0.95, 0.95, 0.96, respectively, which was stable and satisfactory. Figure 6 describe the confusion proportions for each vegetation types of the TP in 2020. The PA showed that the water (14) had the highest accuracy, followed by deciduous broad-leaved forest (4), permanent snow/ice (15), alpine desert (11), alpine grassland (9), deciduous coniferous forest (5), alpine meadow (8), cultivated vegetation (12) in decreasing order. This suggests that vegetation types with distinct characteristics or those that covered a significant portion of the TP generally exhibited relatively high accuracy. However, the accuracy of coniferous and broad-leaved mixed forest (3), evergreen broad-leaved forest (1), evergreen coniferous forest (2), scrub (6), wetland (13), alpine scrub meadow (7), alpine vegetation (10), and non-vegetated area (16) was poor, indicating that complex vegetation types may result in misclassification. For instance, there was notable confusion between alpine scrub meadow and scrub or alpine meadow. Likewise, coniferous and broad-leaved mixed forest is easily confused with evergreen coniferous forest or deciduous broad-leaved forest. Alpine vegetation is susceptible to misclassification due to its high similarity to alpine meadow in reflectance characteristics. The accuracy ranking of UA was similar to that for PA, the permanent snow/ice achieved the highest accuracy.

The performance of the model was verified by using 5-fold cross validation. The training samples were divided into five subsets, and five cross-validation experiments were conducted. In each experiment, one subset was used for validation, while the remaining four subsets were used for training. The final accuracy was calculated by averaging the five accuracies obtained. The overall accuracies for the years 1990, 2000, 2010, and 2020 were 95.02%, 95.42%, 94.93%, and 95.22%, respectively. The corresponding Kappa coefficients were 0.95, 0.95, 0.94, and 0.95, respectively.

### Comparison with sampling sites from literature and field

The results of the vegetation classification were validated using sampling sites from literature and field survey. In 2010, a total of 114 sampling sites of vegetation type were collected from the literature, among which 101 sampling sites were correct, resulting in a validation accuracy of 88.60%. The alpine meadow, achieved an accuracy of 91.84%. In 2020, there were 235 validation sampling sites, the validation accuracy was 83.83%, with 93.97% for alpine meadow and 83.13% for alpine grassland. The validation results indicated that the vegetation maps produced in this study had satisfactory performance.

### Comparison with Landsat imagery

**Fig. 7:**
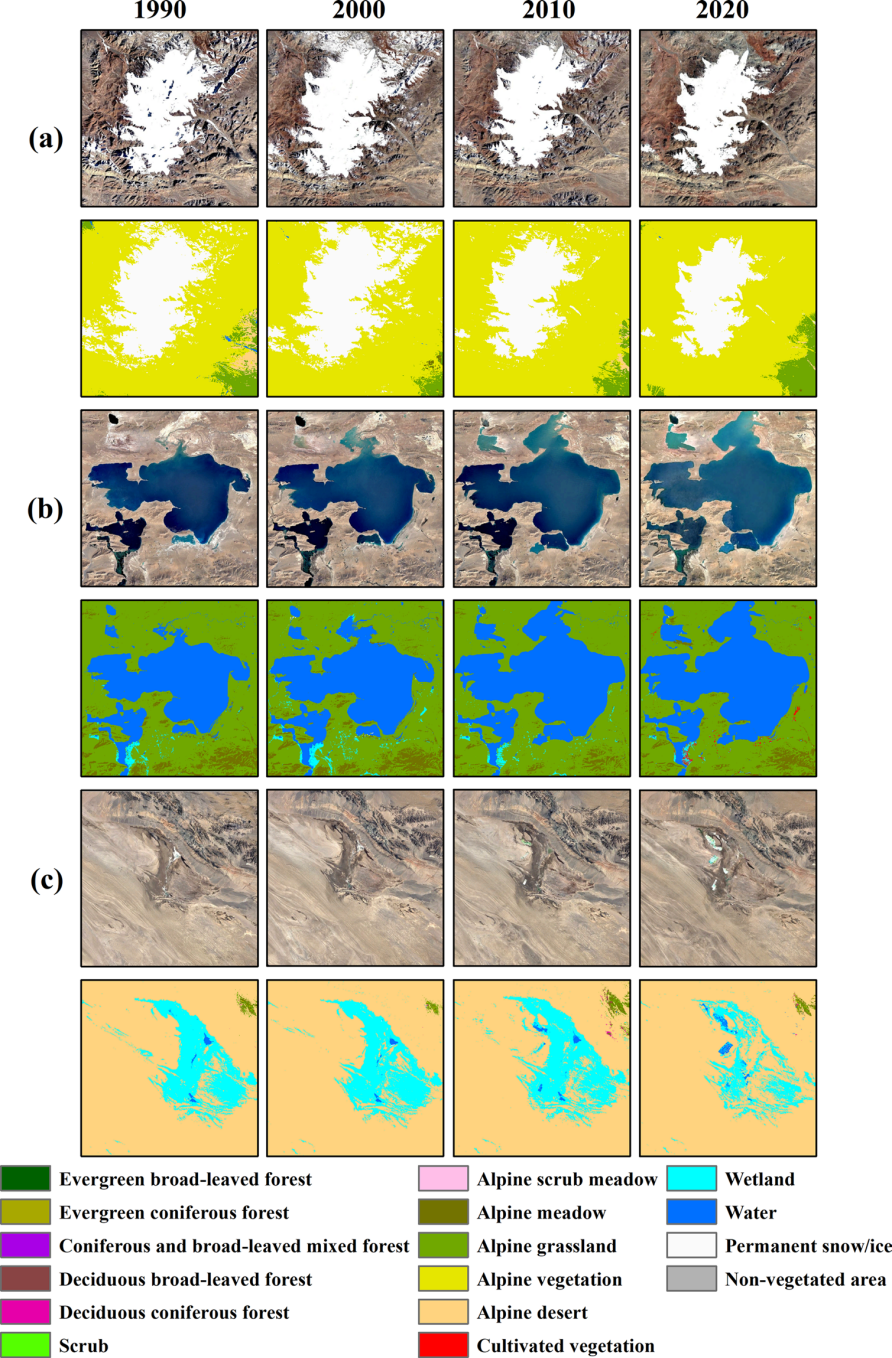
Comparison between the results from this study and Landsat image in details.

We further compared the results with Landsat images. Over the past three decades, the permanent snow/ice is thawing (Fig. 7a), the Siling Co in the southwest of the TP has expanded (Fig. 7b), while the change of wetland in Tsaidam Basin was increased slightly between 1990 and 2000 but decreased after 2000 (Fig. 7c). The results show that there is significant consistency between our classification results and Landsat remote sensing images. Our results can capture the changes in the TP under climate change. This demonstrates the reliability of our data and validates its suitability as foundational data for further research.

### Comparison with previous studies

We intercompared the map of 2020 with the work of Zhou *et al*.^16^ to better reflect its quality. 235 validate sampling sites collected from literature (59) and field survey (176) were used. These validation sampling sites from field survey are considered to have high classification accuracy^28^. The accuracy of Zhou *et al*.^16^ is 0.69, in which alpine meadow, alpine grassland and cultivated vegetation had the highest accuracy, which were 87.50%, 78.45% and 68.67%, respectively. The comparisons demonstrated that the vegetation maps generated in this study exhibited significant strengths in spatial detail and a more extensive variety of vegetation types.

Hu *et al*.^11^ extracted clean glaciers and debris-covered glaciers on the TP from 1985–2020 using a double RF algorithm and Landsat remote sensing imagery. The investigation revealed a discernible recession in the overall glacier extent from 1985 to 2020, with similar decrease trend found in this study. Zhang *et al*.^29^ designated lakes exceeding an area of 1 square kilometer based on Landsat data. The total area of these lakes was found to have expanded from 1976 to 2018. We revealed generally similar trend. Our study has identified a diminishing trend in the wetland situated on the TP, as reported by Li *et al*.^30^ and Xue *et al*.^31^. Some studies treated lake as a subset of wetland to derive results of increased wetland area. However, this is largely due to the fact that the increase in lake area far outweighs the decrease in wetland.

However, at present, these studies mostly focus on permanent snow/ice, lake, or wetland, with limited research on other vegetation types. This study presents a thorough analysis of the vegetation distribution pattern and its dynamic evolution on the TP. It encompasses a broader range of vegetation types and exhibits enhanced accuracy. The observed changing trend aligns with findings from previous research. The outcomes hold paramount significance in enhancing our understanding of the comprehensive alterations in environmental and ecological functionality across the TP.

### Inter-comparison with other products

**Fig. 8:**
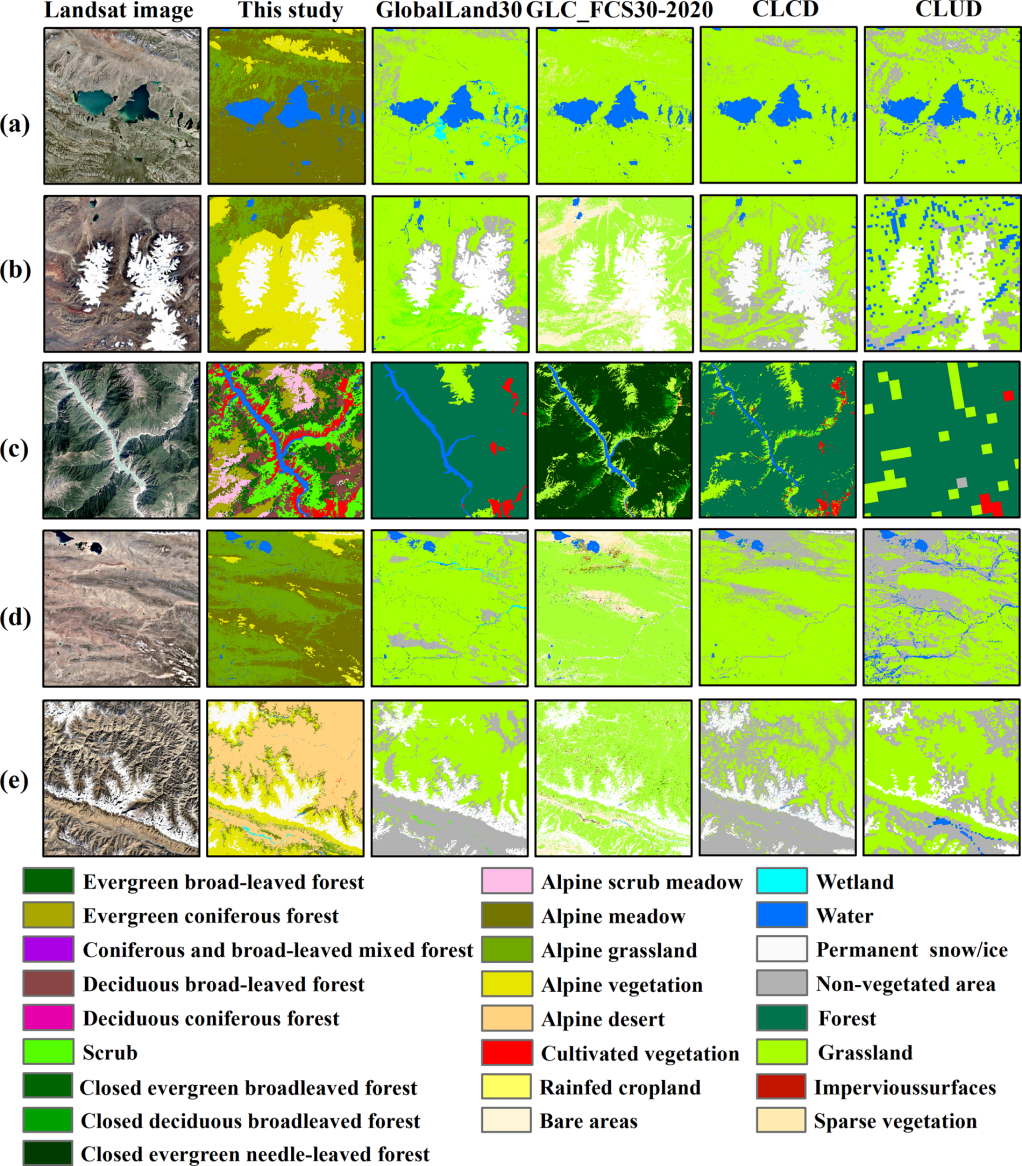
Comparison of our 2020 vegetation map of the TP with other land cover products (GlobeLand30, GLC_FCS30-2020, CLCD, and CLUD) at the first level in five typical sample regions.

We assessed the performance of our maps and 4 landcover products in the TP, as shown in Fig. 8. All of these products were present the vegetation distribution in 2020. GlobeLand30^17^ comprises ten distinct categories, namely water bodies, wetland, permanent snow and ice, artificial surfaces, cultivated land, forest, shrubland, grassland, tundra and bare land. The GLC_FCS30-2020^32–35^ dataset encompasses 29 delineated land cover classifications and there are 26 types in the TP. CLCD^8,36^ has nine categories: cropland, forest, shrub, grassland, water, snow/ice, barren, impervious and wetland, which is similar to GlobeLand30. The first level of CLUD^37–45^ primarily differentiated into cultivated land, forest, grassland, water, built-up areas, and unutilized land, based on the attributes of land resources and their utilization. The second level is mainly divided into 23 types according to the natural properties of land resources. From a classification perspective, GlobeLand30 and CLCD do not further subdivide grassland and forest. However, GLC_FCS30-2020 and CLUD conduct further delineations based on primary classification. GLC_FCS30-2020 provides finer classifications for cultivated land and forest but does not subdivide grassland further. The second level in CLUD is based on forest canopy closure and grassland coverage. In this study, forests are classified into evergreen broad-leaved forest, evergreen coniferous forest, coniferous and broad-leaved mixed forest, deciduous broad-leaved forest, and deciduous coniferous forest. The classification of grassland also takes into consideration the characteristics of alpine ecosystem, subdividing them into alpine meadow, alpine grassland, and alpine desert. Moreover, targeted identification of alpine vegetation is conducted.

We also conducted a simultaneous visual comparison among the 5 products. In Fig. 8, our products demonstrate superior performance compared to others, featuring a finer classification system and enhanced capability in identifying small features. Among the five products, all accurately identify large water bodies such as lakes (Fig. 8a). However, for elongated rivers, the classification results of our study also performed well. In Fig. 8c, it is comparable to GLC_FCS30-2020 and CLCD. Alpine vegetation is widely distributed across various high-altitude regions of the TP, often overlooked in other products. Our study provides comprehensive coverage of alpine vegetation distribution, which holds significant importance for monitoring high-altitude environments in the TP (Fig. 8b). This study classifies forests more precisely (Fig. 8c), and accurately identifies the boundary between alpine meadow and alpine grassland (Fig. 8d). The five products all showed good performance in identifying permanent snow/ice (Fig. 8e).

### Importance of input features

In each year, we select a set of features with the smallest out-of-bag error as the optimal feature subset, and combine four optimal feature subsets to get the final input features. Finally, a set of 27 features is selected for mapping, including Tmp, Pre, elevation, slope, aspect, Tmx, Tmn, Tmp1, Tmp2, Tmp3, Tmp4, Pre1, Pre2, Pre3, Pre4, SAVI_90%, BI_90%, Swir2_15%, BI_30%, NDBI_75%, Blue_15%, Green_15%, NDGI_15%, Green_75%, Blue _30%, Red_15%, IBI_75%.

The results of the feature importance ranking indicated that both climatic factors and terrain factors were more important. As Cheng *et al*.^46^ and Zhang *et al*.^47^ stated, terrain factors are the most important influencing factors in forest/land cover classification of southeast Tibet. This study provides evidence supporting the significant influence of climate factors on the TP, which is worthy of attention in the future research.

## Usage Notes

### Vegetation dynamics in 1990–2020

Based on the vegetation maps, we analysed the vegetation dynamics in the TP from 1990–2020. It is found that the vegetation pattern and composition of TP have been stable for the past 30 years. In a southeast to northwest gradient, a sequential distribution is observed encompassing forest, scrub, alpine meadow, alpine grassland, and alpine desert. To enhance our understanding of its dynamics, we employ the percentage of change area to study variations in each type. It is found that alpine vegetation, wetland, water and permanent snow/ice were the four types that have changed most significantly, as Fig. 7. The overall area in alpine vegetation was 317756.15 km^2^ in 1990 and 357538.2 km^2^ in 2000, and then began to decline, decreasing to 332008.19 km^2^ in 2020, but still an increase of 4.49% compared with 1990. In the context of global warming, the area of permanent snow/ice has been decreasing, and only 114176.46 km^2^ remains in 2020, which represents a decrease of 9.71% relative to that in 1990. At the same time, the total water area showed an increasing trend, increasing by 11.65%, approximately 6700 km^2^. The melting permanent snow/ice, resulting from the global warming, is one of the reasons that account for the water extension. Another reason is the increase in precipitation on the TP in recent decades. The wetland increased slightly by 4.59% between 1990 and 2000 but decreased by 17.92% after 2000.

Concerning the four vegetation types exhibiting the most pronounced alterations in area change, our analysis using the transfer matrix indicates that the increase in alpine vegetation primarily stems from alpine grassland, while the melting of permanent snow/ice predominantly transitions into alpine meadow. The increase of water is mainly from alpine grassland, and the wetland is mainly changed into alpine desert, which leads to its decrease. Despite the relatively modest changes in the transfer matrix for forests and other vegetation, the importance of these changes should not be underestimated. For instance, quantitative assessments by Lu *et al*.^48^ reveal a discernible upward movement of alpine tree lines over the past century. Although the transition from scrub-related categories to forests is minimal within the timeframe of this study, continuous monitoring of changes over extended time series under the influence of ongoing warming is imperative.

### Supplementary information

Supplementary File 1

## Data Availability

In this study, python version of GEE and ArcGIS were used to generate and analyze the result maps. The code used is available on the National Tibetan Plateau/Third Pole Environment Data Center 10.11888/Terre.tpdc.301126^27^.
