# Supplementary material for: The 30 m vegetation maps from 1990 to 2020 in the Tibetan Plateau
Source: Sci Data. 2024 Jul 20;11:804. doi: 10.1038/s41597-024-03649-7 (PMC11271311; doi:10.1038/s41597-024-03649-7)
Supplement: Supplementary file 1 [file 41597_2024_3649_MOESM1_ESM.pdf]

Appendix

1. Dang, Y. Biomass changes of four typical types of alpine grass. *Qinghai Prataculture.* **30(2),** 11-13 (2021).
2. Ding, J., Zhou, G., Chen, K., Chen, S. & Cui, H. Effects of depressions formed by freeze-thaw and water erosion on soil respiration in alpine marsh meadow in Qinghai-Tibet Plateau. *Bulletin of Soil and Water Conservation.* **40(1),** 17-23 (2020).
3. Guo, Q. *et al.* Analysis on the vegetation composition and interspecific association of kobresia meadow in southern pastoral area of southern Qinghai Province. *Acta Agrestia Sinica.* **24(2),** 294-301 (2016).
4. Hu, L., Ade, L., Zi, H. & Wang, C. Effects of plateau zokor disturbance and restoration years on soil nutrients and microbial functional diversity in alpine meadow. *Chinese Journal of Applied Ecology.* **26(9),** 2794—2802 (2015).
5. Jia, Z., Ma, X., Lei, S., Xu, C., Wei, X. & Liu, W. Effects of fertilization on vegetation characteristics of light degraded meadow in Guinan County. *Acta Agrestia Sinica.* **27(4),** 987-996 (2019).
6. Li, C., Zhang, X., Zhao, L., Cheng, G. & Xu, S. Phylogenetic diversity of bacteria isolates and community function in permafrost-affected soil along different vegetation types in the Qinghai-Tibet Plateau. *Journal of Glaciology and Geocryology.* **34(3),** 713-725 (2012).
7. Li, F. *Effects of different management models on the community structure of free-living nitrogen-fixing microbes in the Alpine Meadow soil on Tibet Plateau.* Lanzhou University (2018).
8. Li, S. & Wu, J. Community assembly and functional leaf traits mediate precipitation use efficiency of alpine grasslands along environmental gradients on the Tibetan Plateau. *PeerJ.* **4,** e2680 (2016).
9. Li, T. *Research on soil and vegetation carbon storage of grassland under different land use and degree in Aba Pastoral Areas.* Sichuan Agricultural University (2013).
10. Lin, L. *et al.* The impossible relationships between plant functional groups and organic carbon content under different land utilization types in alpine grassland. *Acta Agrestia Sinica.* **23(1),** 55-61 (2015).
11. Liu, D., Yang, Q., Chen, Z., Zeng, F. & Wei, H. Pollen record and palaeovegetation and palaeoenvironment in Kobresia tibetica alpine wetland. *Journal of Glaciology and Geocryology.* **38(2),** 539-548 (2016).
12. Liu, F., Wu, M., Wei, P., Jia, Y. & Chen, S. Variations of soil microbial biomass carbon and nitrogen in alpine meadow of the Shule River headwater region. *Acta Ecologica Sinica.* **40(18),** 6416-6426 (2020).
13. Liu, S. *Effects of livestock and plateau zokor (Myospalax baileyi) on plant diversity and productivity in alpine meadow.* Lanzhou University (2018).
14. Liu, X. *et al.* Effects of three cushion plants on soil nutrient in alpine areas. *Journal of Gansu Agricultural University.* **46(5),** 91-94+100 (2011).
15. Lu, X., Yan, Y., Fan, J., Cao, Y. & Wang, X. Dynamics of Above- and Below-ground Biomass and C, N, P Accumulation in the Alpine Steppe of Northern Tibet. *Journal of Mountain Science.* **8(6),** 838–844 (2011).
16. Luo, F. *et al.* Effects of grazing intensity on community characteristics and vegetation living states in alpine meadows. *Pratacultural Science.* **38(11),** 2097-2105 (2021).
17. Ma, Z. *The study of soil seed bank in the Alpine Areas on the Eastern Qinghai-Tibet Plateau.* Lanzhou University (2014).

18. Sa, W., An, L. & Sa, W. Changes in plant community diversity and aboveground biomass along with altitude within an alpine meadow on the Three-River source region. *Chin Sci Bull.* **57,** 3573-3577 (2012).
19. She, Y. *et al.* Study on the characteristics and interrelationship of plant community and soil in degraded alpine meadow. *Acta Agrestia Sinica.* **29(S1),** 62-71 (2021).
20. Su, S. *et al.* Study on soil phosphorus nutrient and its regulatory factors during the restoration of degraded grassland in the source region of Heihe River. *Pratacultural Science.* **39(8),** 1562-1570 (2022).
21. Sun, K., Yuan, M., Qu, X. & Luo, D. Slope Differentiation Characteristics of Alpine Meadow Vegetation Community in Sygera Mountain, Tibet. *Journal of Sichuan Agricultural University.* **40(1),** 90-99 (2022).
22. Tang, L. *Effects of phosphorus addition on plant root characteristics and leaf traits in an alpine meadow of the Northwestern Sichuan.* ChinaSouthwest Minzu University. (2020).
23. Wang, X. *et al.* Relationship between soil surface environmental factors and community characteristics of alpine meadow in different desertification stages. *Acta Ecologica Sinica.* **40(19),** 6850-6862 (2020).
24. Xiong, Y., Wu, P., Zhang, H., Cui, L. & He, X. Dynamics of soil water conservation during the degradation process of the Zoigê Alpine Wetland. *Acta Ecologica Sinica.* **31(19),** 5780-5788 (2011).
25. Yang, Y., Bai, R., Li, S., Li, Z., Li, H. &Shen, Y. Responses of species abundance distribution and community similarity to nitrogen and phosphorus additions in a subalpine meadow. *Acta ecologica sinica.* **37(7),** 2290-2299 (2017).
26. Yang, Z. *Effects of improvement measures on the plant and soil of degraded alpinemeadow.* Qinghai University (2017).
27. Yao, X., *Effects of Achnatherum inebrians-Epichloë gansuensis symbiont on plant composition and soil microbial community diversity in rangelands.* Lanzhou University. (2019).
28. Yao, X., Cai, H., Liu, H. & Li, C. Analysis of rumen fermentation characteristics and in vitro digestibility of alpine meadow forage in different seasons. *Acta Agrestia Sinica.* **29(8),** 1729-1737 (2021).
29. Yu, X. *Analysis of spectral characteristics of vegetation in warm steppe and alpine meadow with different utilization and degradation degree.* Capital Normal University. (2012).
30. Zhang, G. *et al.* Responses of key ecological attributes to multi-path restoration measures of degraded alpine meadows. *Acta Ecologica Sinica.* **40(18),** 6293-6303 (2020).
31. Zhang, J. *Effects of nitrogen addition on plant community structure of alpine meadow of Qinghai-Tibetan Plateau.* Lanzhou University. (2011).
32. Zhang, J., Liu, K. & Wang, J. The Relationship between Seed Germinability and Species Abundance in Communities on Eastern Qinghai-Tibet Plateau. *Plant Diversity and Resources.* **35(3),** 317-326 (2013).
33. Zhang, R., Xu, D., Chen, L. & Wang, G. Plant N Status in the Alpine Grassland of the Qinghai-Tibet Plateau: Base on the N:P Stoichiometry. *Environmental Science.* **35(3),** 1131-1137 (2014).
34. Zhang, Y. *Effects of summer and winter rotational grazing of Tibetan sheep on plant, soil and livestock of alpine ecosystem.* Lanzhou University. (2016).
35. Zhang, Y., Li, X., Gao, Z., Zhang, J., Zhou, W. & Zhang, Y. Effects of different fertilization treatments on artificial vegetation and soil microbial characteristics in muli mining area of Qinghai Province. *Acta Agriculturae Boreali-occidentalis Sinica.* **31(6),** 741-754 (2022).

36. Zhao, D. *Effects of short-term grazing rest and grazing prohibition on vegetation characteristics and productivity on alpine meadow.* Lanzhou University. (2020).
37. Zhou, L. *et al.* Simulations of phenology in alpine grassland communities in Damxung, Xizang, based on digital camera images. *Chinese Journal of Plant Ecology.* **36(11),** 1125–1135 (2012).
38. Zi, H. *et al.* Difference of community characteristics and niche of dominant species in different grassland types of alpine meadow. *Chinese Journal of Applied and Environmental Biology.* **22(4),** 0546-0554 (2016).
